# Characterization of Precursor-Dependent Steroidogenesis in Human Prostate Cancer Models

**DOI:** 10.3390/cancers10100343

**Published:** 2018-09-20

**Authors:** Subrata Deb, Steven Pham, Dong-Sheng Ming, Mei Yieng Chin, Hans Adomat, Antonio Hurtado-Coll, Martin E. Gleave, Emma S. Tomlinson Guns

**Affiliations:** 1 Department of Pharmaceutical Sciences, College of Pharmacy, Larkin University, Miami, FL 33169, USA; sdeb@alumni.ubc.ca; 2 The Vancouver Prostate Centre at Vancouver General Hospital, 2660 Oak Street, Vancouver, BC V6H 3Z6, Canada; steven7801z@gmail.com (S.P.); dsming@hotmail.com (D.-S.M.); mchin@prostatecentre.com (M.Y.C.); hadomat@prostatecentre.com (H.A.); ahurtado@prostatecentre.com (A.H.-C.); m.gleave@ubc.ca (M.E.G.); 3 Department of Urologic Sciences, Faculty of Medicine, University of British Columbia, Vancouver, BC V5Z 1M9, Canada

**Keywords:** castration resistant prostate cancer, steroidogenesis, cell line, prostate tissue metabolism, liquid chromatography-mass spectrometry, backdoor pathway

## Abstract

Castration-resistant prostate tumors acquire the independent capacity to generate androgens by upregulating steroidogenic enzymes or using steroid precursors produced by the adrenal glands for continued growth and sustainability. The formation of steroids was measured by liquid chromatography-mass spectrometry in LNCaP and 22Rv1 prostate cancer cells, and in human prostate tissues, following incubation with steroid precursors (22-OH-cholesterol, pregnenolone, 17-OH-pregnenolone, progesterone, 17-OH-progesterone). Pregnenolone, progesterone, 17-OH-pregnenolone, and 17-OH-progesterone increased C21 steroid (5-pregnan-3,20-dione, 5-pregnan-3,17-diol-20-one, 5-pregnan-3-ol-20-one) formation in the backdoor pathway, and demonstrated a trend of stimulating dihydroepiandrosterone or its precursors in the backdoor pathway in LNCaP and 22Rv1 cells. The precursors differentially affected steroidogenic enzyme messenger RNA (mRNA) expressions in the cell lines. The steroidogenesis following incubation of human prostate tissue with 17-OH-pregnenolone and progesterone produced trends similar to those observed in cell lines. Interestingly, the formation of C21 steroids from classical pathway was not stimulated but backdoor pathway steroids (e.g., 5-pregnan-3,20-dione, 5-pregnan-3-ol-20-one) were elevated following incubations with prostate tissues. Overall, C21 steroids were predominantly formed in the classical as well as backdoor pathways, and steroid precursors induced a diversion of steroidogenesis to the backdoor pathway in both cell lines and human prostate tissue, and influenced adaptive steroidogenesis to form C21 steroids.

## 1. Introduction

Development and progression of prostate cancer (PCa) primarily depend on androgens. Testosterone is typically generated by Leydig cells in the testes, and it circulates to peripheral target tissues [1,2]. While surgery or radiation offer curative solutions to organ-confined diseases, androgen deprivation therapy (ADT) remains the first line of treatment in men with advanced metastatic PCa. In response to ADT, testosterone production is significantly suppressed, resulting in the regression of the tumor. However, ADT is not curative, and PCa often progresses to castration-resistant prostate cancer (CRPC) [3,4]. During CRPC, the cancer can be either dependent or independent of androgens. There are multiple mechanisms involved in the development of the androgen-independent phenomenon that enables the cancerous cells to activate the androgen receptor (AR) in the absence of androgens, or recruit other receptors to facilitate cancer progression. Examples of androgen-independent prostate cancer mechanisms include promiscuous AR, hypersensitive AR, splice variant AR, outlaw pathways (e.g., insulin growth factor 1), bypass pathways (e.g., receptor tyrosine kinases), lurker cell pathways, and steroidogenesis [3,5,6,7,8,9]. Previous reports have shown that prostate tumors have increased levels of steroidogenic enzymes that synthesize androgens from cholesterol or other circulating steroid precursors, such as progesterone or dehydroepiandrosterone (DHEA) [3,10,11,12]. As such, intratumoral steroidogenesis that takes place in prostate cancer cells has been identified as a castration resistance mechanism where AR is reactivated despite low levels of circulating testosterone [2,3].

Steroidogenesis is the term used for the multistep process, either via the “classical” pathway or the “backdoor” pathway, that involves the conversion of cholesterol to a comprehensive array of downstream steroids, including testosterone and dihydrotestosterone (DHT) through multiple steroidogenic enzymes [3,13]. The classical pathway involves the conversion of cholesterol to pregnenolone by steroidogenic acute regulatory protein (STAR) and cytochrome P450 11A1 (CYP11A1). Pregnenolone is subsequently converted to progesterone by hydroxy-delta-5-steroid dehydrogenase 3B1 (HSD3B1) or HSD3B2. CYP17A1, along with cytochrome b5 (CYPB5), converts pregnenolone and progesterone to DHEA and androstenedione, respectively. DHEA can be converted to androstenedione by HSD3B1 or 2, and androstenedione is converted to testosterone by hydroxysteroid 17-beta dehydrogenase 3 (HSD17B3) or aldo-keto reductase 1C3 (AKR1C3), and subsequently to DHT by steroid 5-alpha-reductase type 1 (SRD5A1) or 2 isoforms. DHEA can also be converted to androstenediol by HSD17B3 or AKR1C3 and then to testosterone by HSD3B1 or HSD3B2 [2,3]. In contrast, the backdoor pathway produces DHT without generating testosterone. SRD5A1 catalyzes the formation of 5-pregnan-3,20-dione or 5-pregnan-17-ol-3,20-dione from progesterone or 17-OH-progesterone as starting materials. These downstream substrates are subsequently converted to 5-pregnan-3-ol-20-one and 5-pregnan-3,17-diol-20-one, respectively, by AKR1C2. CYP17A1 acts on either substrate to generate androsterone. AKR1C3 converts androsterone to androstanediol, which can ultimately be converted to DHT via the action of HSD17B6. Alternatively, androstenedione can be converted to androstanedione by SRD5A1 and subsequently to DHT by HSD17B3 [2,3,14]. Variants of steroidogenic enzymes can also influence prostatic steroidogenesis, disease progression, and treatment outcomes. For example, the HSD3B1(1245A>C) germline variant increases the formation of DHT from extragondal substrates and can also compromise the effect of abiraterone, a CYP17A1 inhibitor [15]. Typically, testosterone is generated from the testes for circulation throughout the body [1,2]. In the prostate gland, the classical steroidogenesis pathway is somewhat functional during the early stages of PCa. Under castrated conditions, circulating testosterone is significantly suppressed; however, the adrenal glands can act as a source of DHEA as well as progesterone, which can be used by the prostate tumor to generate testosterone and DHT. The backdoor pathway along with the classical steroidogenic reactions are active during CRPC condition. The steroidogenic enzymes required for the synthesis of testosterone and DHT become upregulated in CRPC to enhance the turnover of circulating precursors, as well as cholesterol for androgen production in the tumor [1,2,3]. SRD5A1 enzyme levels are often overexpressed in CRPC, which has a critical role in the backdoor pathway to generate DHT [3,16]. In contrast, SRD5A2 levels appear to be decreased during the advanced stages of prostate cancer [12,17,18]. The adaptive ability of PCa cells to produce steroids ultimately implies a significant advantage to grow and resist treatment.

Various models, including cell lines, xenograft tissues and human tissues, have been used to study steroidogenesis in CRPC. Cell lines such as LNCaP, 22Rv1, PC3, and others are relatively easy to culture and to conduct these experiments with. However, the potential to generate steroids varies among cell lines because of the differences in enzyme expression and steroid metabolism in these cell lines [10,16]. LNCaP prostate cancer cells were originally derived from human metastatic lesions in lymph nodes, and they have an active AR and respond to androgen stimulation (androgen-dependent) [19,20]. It has been demonstrated that although LNCaP cells show varying levels of steroidogenic enzymes depending on their passage number and growth environment, they express the full complement of enzymes that are necessary for the conversion of cholesterol to testosterone [10,21,22,23]. In comparison, 22Rv1 cells were originally derived from CWR22 human prostate cancer xenografts under castrated conditions in mice [24]. These cells underwent several adaptations growing under steroid suppressed environments, leading to the development of spliced AR variants that are activated in the absence of androgen ligands (androgen-independent) [25,26]. Xenograft models are more complex than the cell line models, and they are likely to reflect the influences of the tumor microenvironment and systemic factors on steroidogenesis. Studies using human tissues are even more complicated due to prior treatment regimens, the heterogeneity of tumors, diet, and other factors that can direct tumor development and cancer progression, not to mention challenges faced in sourcing material for research. Furthermore, radioactive-labelled precursors and high performance liquid chromatography (HPLC) with in-line radiometric detection have previously been used to determine steroid formation in these models. In recent years, there has been a preference to use liquid chromatography-mass spectrometry (LC/MS), due to its ease and sensitivity. However, very limited LC/MS data are available on prostate cellular or tissue steroidogenesis.

In the current study, we have compared the LNCaP (androgen-dependent) and 22Rv1 (androgen-independent) cells alongside human prostate tissues in vitro for their steroidogenic abilities following incubation with upstream precursors of testosterone and DHT, such as 22-OH-cholesterol, progesterone, pregnenolone, 17-OH-progesterone, or 17-OH-pregnenolone. It has been demonstrated that although LNCaP cells show varying levels of steroidogenic enzymes depending on their passage number and growth environment, they express the full complement of enzymes necessary for the conversion of cholesterol to testosterone [10,21,22,23,27]. 22Rv1 cells have demonstrated high levels of steroidogenic enzymes and the ability to form testosterone and DHT using upstream precursors [10,28]. The precursors can affect various enzyme levels, adaptations between the classical and backdoor pathway, and androgen metabolism. Therefore, the current study exemplifies how different cell lines and substrates modulate the steroidogenesis profile for use in prostate cancer research. For the purpose of this work, a comparison of steroidogenesis between human prostate tissue and cell lines was completed in order to explore a more representative model of prostate steroidogenesis in vivo.

## 2. Results

### 2.1. Quantification of Steroid Formation by LC/MS

**Figure 1:**
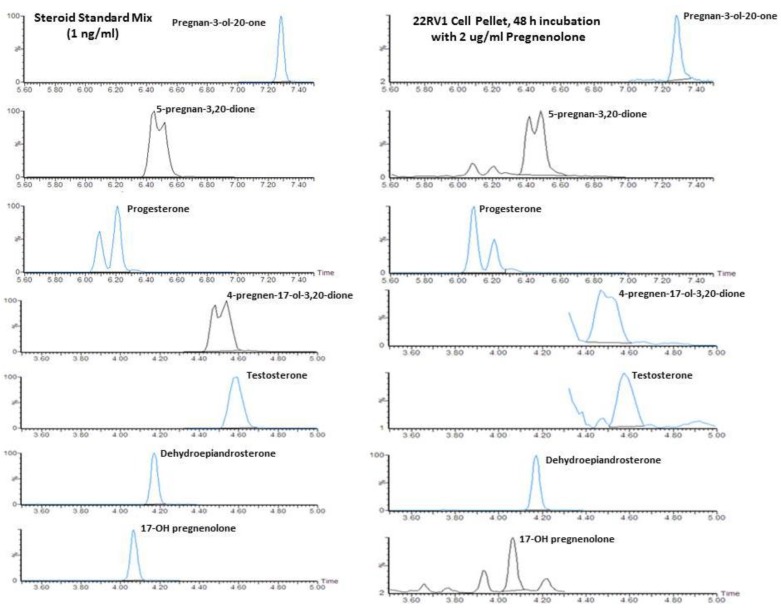
Representative liquid chromatography-mass spectrometry (LC/MS) chromatograms showing steroid standards (1 ng/mL) and the formation of steroids following the incubation of 22Rv1 cells with 2 μg/mL pregnenolone for 48 h (measured in the cell pellet).

A representative chromatogram obtained for 22Rv1 incubated with 2 µg/mL pregnenolone and calibration standard is presented in Figure 1 for 5-pregnan-3-ol-20-one, 5-pregnan-3,20dione, progesterone, 17-OH-progesterone (4-pregnen-17-ol-3,20-dione), testosterone, DHEA, and 17-OH-pregnenolone. Similar chromatograms were also observed for incubation without any precursor when sufficient cellular material or media was available, in order to meet lower detection limits of 0.01 ng/mL. The internal standards, including trideuterated testosterone (d3T) and trideuterated dihydrotestosterone (d3DHT), provided very good matrix correction for testosterone and DHT, and they were also reasonable for the other ketosteroids profiled with the oxime derivatization. The 5α-androstan-3β,17β-diol-16,16,17-d3 (d3diol) was not used to quantify the hydroxylamine-derivatized oxime steroids, as unlike a ketosteroid, it is not converted to an oxime. However, d3diol was present in the internal standard mixture for fluoromethypyridinium derivitization of hydroxy steroids, another assay used in our laboratory.

### 2.2. Effects of Precursor Stimulation on the Steroidogenesis Pathway in the In Vitro Models

Treatment of 22Rv1 cells with 22-OH-cholesterol had very minimal effects on the steroid pathway in the cells (Table 1). Only a statistically significant decrease was observed for androstenedione when compared to the control (Table 1). No observable differences were found for steroids in the media (Table S2). Treatment with pregnenolone increased DHEA, progesterone, 5-pregnan-3,20-dione, 17-OH-pregnenolone, and 5-pregnan-3-ol-20-one levels when compared to the control, and were found to be statistically significant (Table 1). There was a statistically significant decrease in testosterone levels compared to the control (Table 1). In the media, DHEA, progesterone, 5-pregnan-3,20-dione, 17-OH-pregnenolone, and 5-pregnan-3-ol-20-one were increased when compared to the control (Table S2). Treatment of the cells with progesterone produced statistically significant increases in DHEA, 17-OH-progesterone, pregnenolone, 5-pregnan-3,20-dione, and 5-pregnan-3-ol-20-one when compared to the control (Table 1). Progesterone treatment also produced a statistically significant decrease in testosterone levels. In the media, increases in androsterone and pregnenolone, and 17-OH-pregnenolone were found to be statistically significant (Table S2). Following treatment with progesterone, unlike vehicle-treated cells, androstenedione was also detected. When treated with 17-OH-pregnenolone, the increase of 17-OH-progesterone was found to be statistically significant in the cells and media (Table 1 and Table S2). DHEA was produced in high amounts but was found to be statistically significant only in media. 5-Pregnan-3,17-diol-20-one was found to be moderately increased in the media and increased much more in cells. 17-OH-progesterone treatment produced statistically significant increases in androsterone and pregnenolone in the cells (Table 1). In the media, 17-OH-progesterone stimulated the formation of androstenedione and testosterone which were not present in vehicle-treated samples (Table S2). 5-Pregnan-3,17-diol-20-one was produced in large amounts both in the media and cells (Table 1 and Table S2). The levels of the steroids (22-OH-cholesterol, pregnenolone, 17-OH-pregnenolone, progesterone, 17-OH-progesterone) that were incubated with the cells exceeded the dynamic range of detection, and thus they could not be quantified accurately in their respective incubations.

Treatment of LNCaP cells with 22-OH-cholesterol produced no significant increases or decreases in either cells or media. Treatment with pregnenolone increased 5-pregnan-3,20-dione and 17-OH-pregnenolone in the cells (Table 2). Similarly, progesterone was produced in large amounts following incubation with pregnenolone. In the media, an increase in progesterone was found to be statistically significant when compared to the control (Table S3). DHEA was found to be increased in cells by almost two-fold compared with the control, however this was not found to be statistically significant. DHEA had a statistically significant increase in the media. When treated with progesterone, increases in pregnenolone and 5-pregnan-3,20-dione were found to be statistically significant in the cells when compared to the control (Table 2). In the media, increases in DHEA, pregnenolone, and 5-pregnan-3,20-dione were found to be statistically significant (Supplemental Table S3). 5-Pregnan-3-ol-20-one was produced in cells and media, and it was not detected in the vehicle-treated controls (Table 2 and Table S3). When LNCaP cells were treated with 17-OH-pregnenolone, statistical significant increases were found for DHEA in the cells and DHEA, and pregnenolone in the media (Table 2 and Supplemental Table S3). Similarly, following incubation with 17-OH-pregnenolone, 17-OH-progesterone was produced in high amounts, but it was not detected in the vehicle-treated control. Treatment with 17-OH-progesterone produced statistically significant increases in testosterone, androsterone, pregnenolone, and 17-OH-pregnenlone in the cells (Table 2). In the media, 17-OH-progesterone increased DHEA, androsterone, pregnenolone, and progesterone levels (Supplemental Table S3). Likewise, following incubation with 17-OH-progesterone, the formation of testosterone increased both in the LNCaP cell pellet and the media, but it was either not detected (media) or had very low levels (cell pellet) in the vehicle-treated control. 

**Figure 2:**
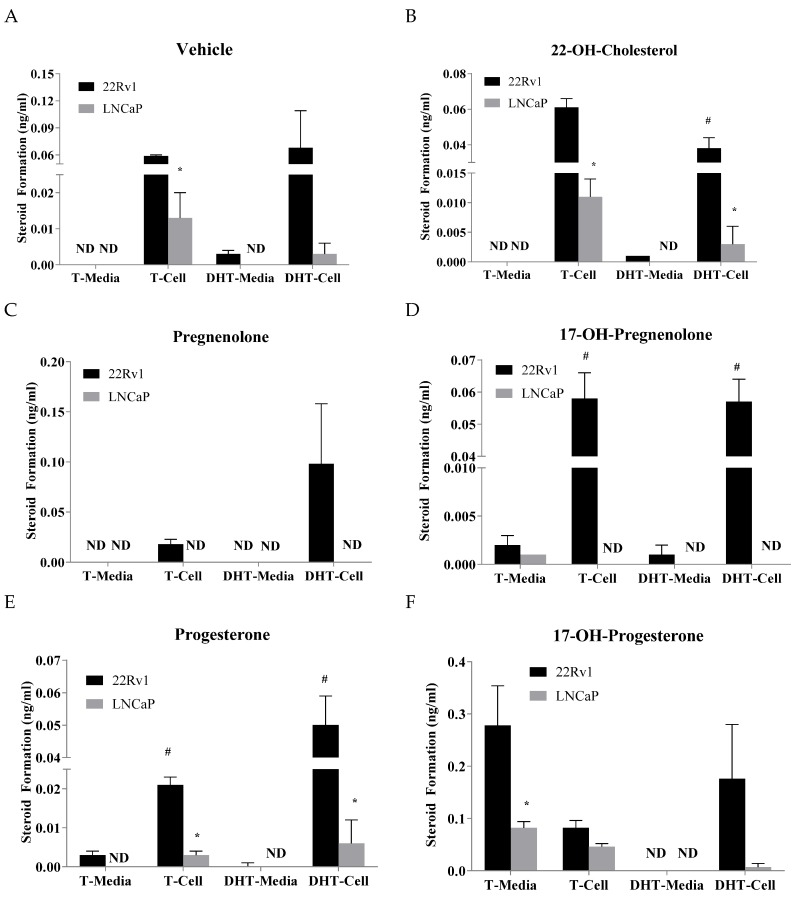
Testosterone (T) and dihydrotestosterone (DHT) levels (ng/mL) in LNCaP and 22Rv1 cell culture media and pellet, following incubation with 2 μg/mL 22-OH-cholesterol (panel **B**), pregnenolone (panel **C**), 17-OH-pregnenolone (panel **D**), progesterone (panel **E**), 17-OH-progesterone (panel **F**) or vehicle (panel **A**) for 48 h. * represents statistically significant difference (*p* < 0.05) between 22Rv1 and LNCaP cells when comparing either T or DHT in either cell pellet or media. # represents statistically significant difference (*p* < 0.05) between cell pellet and media in a single cell line for either T or DHT. Mean ± SEM values were obtained from three separate experiments performed on different days.

A comparison on how the precursors affect testosterone and DHT levels between LNCaP and 22Rv1 in both the media and cells was carried out. Without any precursor, 22Rv1 cells produced more testosterone and DHT than the LNCaP cells (Figure 2A). There are statistically significant differences found for testosterone in the cells (Figure 2A). When treated with 22-OH-cholesterol, there are statistically significant differences in the testosterone and DHT levels between 22Rv1 and LNCaP cells (Figure 2B). Pregnenolone treatment produced detectable testosterone and DHT only in 22Rv1 cells (Figure 2C). Treatment with 17-OH-pregnenolone produced testosterone and DHT in both the 22Rv1 cells and the media (Figure 2D). However, the levels found in the media were very low. Testosterone was only barely detectable in LNCaP cell media (Figure 2D). Statistically significant differences were found between 22Rv1 cells and media for testosterone and DHT levels. Progesterone treatment produced significantly higher levels of testosterone and DHT in the 22Rv1 than LNCaP cells (Figure 2E). 17-OH-progesterone treatment produces higher levels of testosterone in 22Rv1 cells and media, but only the media was found to be statistically significantly different compared to the LNCaP cells (Figure 2F). Although DHT levels were found to be higher in 22Rv1 than LNCaP cells, they were not found to be statistically significant (Figure 2F).

### 2.3. Effects of Precursors on Steroidogenic Enzymes in the In Vitro Models

In 22Rv1 cells, treatment with 22-OH-cholesterol resulted in a statistically significant downregulation of *SRD5A1* (Figure S1C). Progesterone treatment caused a statistically significant downregulation of aldo-keto reductase 1C1 (*AKR1C1)* when compared to the control (Figure S1A). Treatment with 17-OH-pregnenolone resulted in the statistically significant downregulation of *AKR1C1* and aldo-keto reductase 1C3 (*AKR1C3)* (Figure S1A,B). 17-OH-progesterone produced a statistically significant downregulation of *AKR1C1*, *AKR1C3*, and *HSD17B3* (Figure S1A,B,D). Furthermore, a significant increase of *HSD17B6* messenger RNA (mRNA) levels was found in 22Rv1 cells treated with 17-OH-progesterone (Figure S1E). Treatment of LNCaP cells with 22-OH-cholesterol produced no statistically significant differences in the mRNA levels of the steroidogenic enzymes when compared to the control (Figure S2). Pregnenolone treatment caused a statistically significant downregulation of *HSD17B3* and *STAR* when compared to the control (Figure S2C,D). Progesterone treatment resulted in statistically significant downregulation of *AKR1C3*, *HSD17B3*, and *STAR* when compared to the control (Figure S2A,C,D). A statistically significant upregulation of *SRD5A1* was also observed in LNCaP cells treated with progesterone (Figure S2B). 17-OH-pregnenolone treatment resulted in a downregulation of *AKR1C3*, *HSD17B3*, and *STAR* (Figure S2A,C,D). An upregulation in *SRD5A1* mRNA level was also detected in 17-OH-pregnenolone-treated LNCaP cells (Figure S2). 17-OH-progesterone treatment upregulated *SRD5A1*, and downregulated *HSD17B3* and *STAR* when compared to the control (Figure S2B,C,D). CYP17A1 and SRD5A2 could not be reliably detected in either cell line.

A comparison between LNCaP and 22Rv1 cells can also be made to determine the relative amount of steroidogenic enzyme mRNA in each cell line. Steroid enzyme mRNA data were normalized to housekeeping *GAPDH* mRNA levels, which were assumed to be at the same level per cell, and for both cell lines. Data in Table 3 were represented in fold changes relative to LNCaP for each precursor. In 22Rv1 cells, *AKR1C1* and *AKR1C3* mRNA levels were very high, ranging from a 18–60 fold increase compared to those in LNCaP cells, and they were found to be statistically significant for all precursors used in this study. *HSD3B2*, *HSD17B3*, *HSD17B6*, *RDH5*, and *STAR* levels were also found to be significantly higher (4–40 times) in 22Rv1 than those in LNCaP. Surprisingly, 22Rv1 cells showed overall lower *SRD5A1* mRNA levels than LNCaP, and these levels were significantly lower with progesterone and 17-OH-progesterone treatments.

### 2.4. Effects of 17-OH-Pregnenolone and Progesterone on Steroid Metabolism in Human Peripheral Zone Prostate Tissue

**Figure 3:**
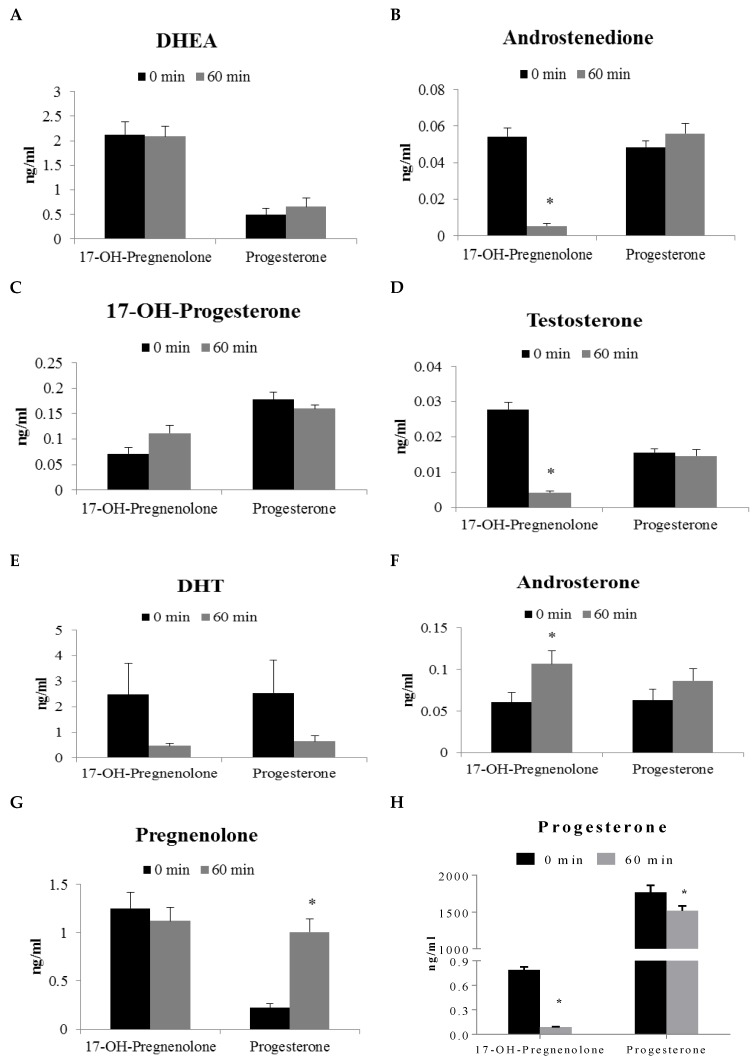

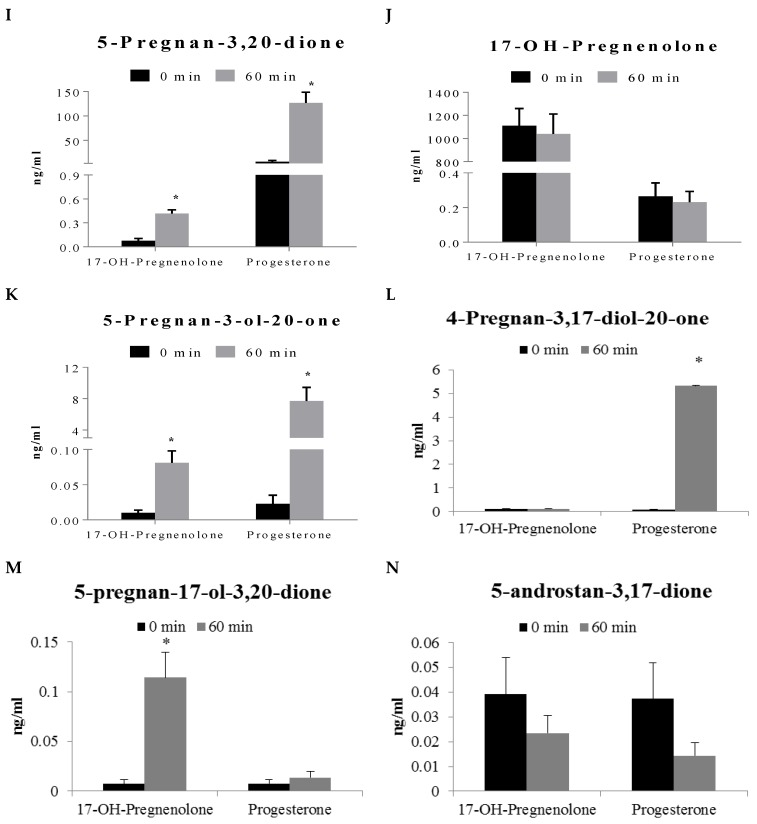
Steroid formation (ng/mL) in human prostate tissue (peripheral zone, *N* = 9) homogenate following 60 min incubation with 2 μg/mL progesterone and 17-OH-pregnenolone precursors. Each of these panels represents the levels of a particular steroid formation. Results are expressed as mean ± SEM of nine different tissues. * represents statistically significant difference (*p* < 0.05) between 0 and 60 min incubations of human prostate homogenate with either 17-OH-pregnenolone or progesterone.

17-OH-Pregnenolone and progesterone were used to investigate steroid metabolism in human prostate peripheral zone tissue. Incubation with 17-OH-pregnenolone resulted in a statistically significant decrease of androstenedione, testosterone and progesterone levels when compared to the 0 min control (Figure 3B,D,H). Androsterone, pregnan-3-20-dione, 5-pregnan-3-ol-20-one, and 5-pregnan-17-ol-3,20-dione were found to be statistically significantly increased after 60 min of 17-OH-pregnenolone incubation (Figure 3F,I,K,M). Progesterone incubation produced statistically significant increases in pregnenolone, 5-pregnan-3,20-dione, 5-pregnan-3-ol-20-one, and 4-pregnan-3,17-diol-20-one when compared to the 0 min control (Figure 3G,I,K,L).

## 3. Discussion

Intratumoral steroidogenesis is a recognized feature of castration-resistant prostate cancer that emerges during cancer progression and treatment resistance. By utilizing circulating steroid precursors or by synthesizing them from cholesterol through steroidogenesis, prostate tumors continue to grow despite drug intervention (chemical castration). It is still unclear how the pathway adapts to new treatment regimens, and whether it is consistent across all cell models and patients. Cell lines and human tissues have differential levels of steroid enzymes, and thus different potentials for steroidogenesis. In the current study, we investigated how the steroid substrates are utilized via the classical and backdoor pathways when incubated with different upstream precursors, namely 22-OH-cholesterol, pregnenolone, progesterone, 17-OH-pregnenolone, and 17-OH-progesterone. We also endeavored to identify the differences between LNCaP and 22Rv1 cell lines alongside human prostate tissue with respect to their steroidogenesis potentials. Since 20-OH cholesterol had relatively low turnover to downstream androgens, it is likely that the side chain cleavage enzyme (CYP11A1) has limited functionality in these prostate cancer cell lines.

**Figure 4:**
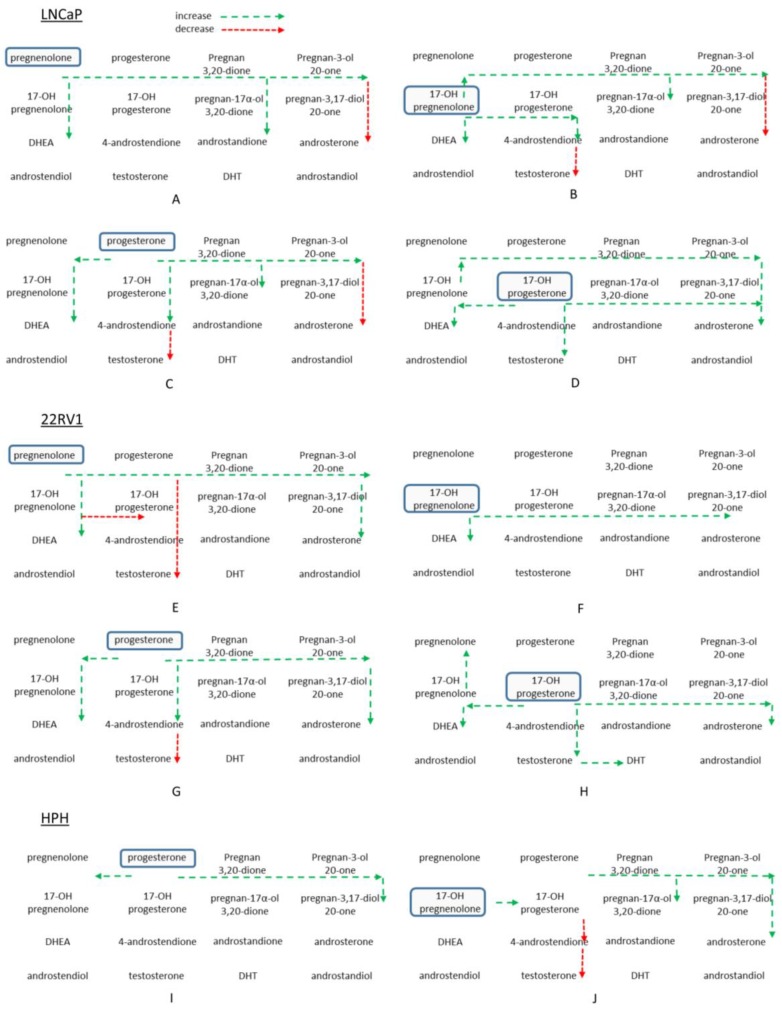
Overview of the relative changes in androgen pathway steroids in vitro following incubation with four different precursors. Panels **A**–**D** are from LNCaP cell pellet extracts after treatment with 2 μg/mL pregnenolone, 17-OH-pregnenolone, progesterone, and 17-OH-progesterone, respectively, for 48 h. Panels **E**–**H** are from 22Rv1 cell pellets with the same treatment and order as for LNCaP cells above. Panels **I** and **J** highlight incubations of 2 μg/mL progesterone and 17-OH-pregnenolone with human prostate homogenate for 60 min. Long dashed green arrows indicate increase, short dashed red arrows indicate decrease.

The pregnenolone, progesterone, 17-OH-pregnenolone, and 17-OH-progesterone steroid precursors facilitated the production of metabolites immediately downstream such as DHEA, which is an important precursor in the classical steroid pathway because it can be readily converted to testosterone and DHT. In fact, DHEA is an alternate precursor source for steroidogenesis that is generated by the adrenal glands. DHEA accumulated in 22Rv1 cells and media, more so when cells were incubated with steroid precursor pregnenolone or 17-OH-pregnenolone. It is conceivable that DHEA produced within the cells is used for androgen production or secreted to other tumor cells for the same purpose. Testosterone and DHT are important for AR activation and the sustained growth of prostate cancer. The precursors used in the study had inconsistent effects on testosterone and DHT production. Progesterone and pregnenolone decreased testosterone and DHT production in 22Rv1 and LNCaP cells. 17-OH-pregnenolone and 17-OH-progesterone either maintained or stimulated testosterone production in 22Rv1 cells. Only 17-OH-progesterone stimulated testosterone production in LNCaP cells. DHT was reliably produced by 22Rv1 cells; however, addition of these precursors had minor effects on the DHT levels. LNCaP cells had nearly undetectable levels of DHT, which leads us to suggest that DHT formation from upstream steroids is highly controlled and, as the Figure 4 overview summarizes, upstream precursors could be redirected to DHEA and the backdoor pathway, thus limiting the precursors from further conversion to androgens. In our previous work, inhibition of steroidogenesis with abiraterone acetate and dutasteride caused an accumulation of steroid upstream precursors [28]. Abiraterone diverted upstream precursors towards the backdoor pathway, possibly due to the accumulation of progesterone and pregnenolone [28]. Dutasteride prevented the use of the backdoor pathway, and caused steroids to accumulate within the classical pathway [28]. When both inhibitors were used in combination, upstream precursors accumulated, and backdoor pathway steroids were produced [28]. The accumulation of precursors can potentially affect steroid enzyme levels and alter how upstream precursors are used to generate downstream androgens. Also, the work by Chang et al., (2011) demonstrated that CRPC cells and fresh tissues can avoid testosterone while synthesizing DHT [16]. This alternative pathway converts androstenedione, which is catalyzed by SRD5A1 enzyme, to 5α-androstanedione and finally 17β-HSD-mediated formation of DHT. Considering the low CYP17A1 activity and the limited formation of C19 steroids in the backdoor pathway, it is conjectured that C21 steroids such as 17-OH progesterone may bypass testosterone and form DHT via 5α-androstanedione. Overall, however, the lower formation of testosterone and DHT from C21 steroids suggests that the CYP17 enzyme has limited activity in those cell lines.

Preference for steroid synthesis via the backdoor pathway is observed when cells are incubated with pregnenolone, progesterone, 17-OH-pregenenolone, and 17-OH-progesterone. In 22Rv1 and LNCaP cells, stimulation with pregnenolone and progesterone increased 5-pregnan-3,20-dione and 5-pregnan-3-ol-20-one levels in both cells and media (Figure 4). Pregnan-17-ol-3,20-dione and pregnan-3-17-diol-20-one are increased by 17-OH-pregnenolone and 17-OH-progesterone in 22Rv1 and LNCaP cells. The backdoor pathway may be one possible way for cells to store precursors for future conversion to DHT (Figure 4). The substrate levels were not accurately quantifiable with this assay without dilution into the dynamic range of measurement, so the depletion of substrates may not be considered significant. Unfortunately, baseline synthesis of steroids within these systems is quite limited, and thus difficult to study. The concentration of the precursor steroid (2 ug/mL) used is supraphysiological, however, due to the analytical challenges this range of concentration is required. The turnover of substrates also appears to be quite limited, despite using large concentrations of steroid precursors. Although previous work from our laboratory has demonstrated that both LNCaP and 22Rv1 cell lines are steroidogenic in nature [28,29], their metabolic capacity to produce the whole range of steroids appears to be low. It may be possible that steroid enzyme levels are not high enough to produce large amounts of androgens under the growth and incubation conditions of the cells in our study. It is important to recognize that the current study includes only two cell lines and nine prostate cancer patient samples. The experimental components, such as variability in cell culture conditions (e.g., growth factors, culture media, passage number) and interindividual biological variability, may have influenced the observed results. Future studies with standardized incubation conditions between different cell lines and a larger patient pool will underscore the outcomes of the current study. It is also possible that androgen production is tightly regulated and prevents a large production of androgens in the presence of high levels of precursors. Glucuronidation of downstream androgens can also be assessed to determine how these androgens are being inactivated under high concentrations of different precursors. It is plausible that the steroids that are produced are conjugated through glucuronidation or sulfation, leading to a lower estimation of steroid production based on the detection of unconjugated steroids only. Also, the precursors used in the experiments are biased towards the conversion of C21 to C19 steroids within prostate cells and tissues. Despite these potential limitations, the present work provides a snapshot of steroidogenesis in prostate cancer cells and tissues.

In the present study, we also observed a substantial and consistent increase in pregnenolone levels in the cell pellet and media following incubation of LNCaP and 22Rv1 cells with progesterone, 17-OH-progesterone, or 17-OH-pregnenolone. However, currently, there is no known pathway that can facilitate this conversion. The presence of an unknown enzyme that can catalyze the conversion of downstream steroids to pregnenolone is plausible. Alternatively, the incubation of a supraphysiological concentration of substrate may have arrested the pathway at pregnenolone such that it accumulates consequently in the cells and media. Many factors clearly influence the steroidogenic capacity of prostatic tissue, and further work is required to unravel these.

The upstream precursor steroids predominantly alter the steroidogenesis pathway and influence the formation of select steroids in the classical and backdoor pathways. The steroidogenic enzyme level changes following incubation with steroid precursors reflect changes to the steroidogenesis pathway. *HSD17B3* and *AKR1C3* levels decrease, which impedes the conversion of androstenedione to testosterone, thus increasing upstream precursors. Increased *SRD5A1* levels are critical for the direct conversion of upstream precursors through the backdoor pathway, as well as through the androstenedione pathway. 17-OH-pregnenolone stimulates DHEA production, as well as the other precursors in the backdoor pathway, by its conversion to 17-OH-progesterone, and then to pregnan-17-ol-3,20-dione. 17-OH-progesterone stimulation produces pregnan-3-17-diol-20-one more readily than 17-OH-pregnenolone. This may be due to the steroidogenic enzyme levels, or the differential preference of precursor utilization. 17-OH-progesterone was better at stimulating testosterone and DHT production than the other precursors, despite decreasing *AKR1C3* and *HSD17B3* enzyme levels. Furthermore, testosterone is secreted into the media in relatively large amounts after 17-OH-progesterone stimulation. Therefore, an increase of 17-OH-progesterone in a tumor due to inhibition of the steroid pathway or upregulation of steroidogenesis as a result of ADT may consequently induce testosterone production and secretion to stimulate the surrounding tumor cells. It is still unclear how these precursors affect changes in the steroid enzyme levels. There may be some interactions between the precursors as weak agonists and the AR (wild type or mutated) to facilitate the changes within the steroidogenesis pathway [30,31,32]. Downstream steroids such as DHEA may also interact with the AR and cause alterations of the enzyme levels within the steroidogenesis pathway [33,34]. Ultimately, androgen production is strictly regulated and steroids are stored as precursors in the backdoor pathway or as DHEA in the classical pathway. Storage in these forms can provide readily available material for the production of testosterone or more importantly DHT [14,35].

In our study, we compared the steroidogenic potential between 22Rv1 and LNCaP prostate cancer cell lines. We observed a greater rate of formation of C21 steroids in 22Rv1 than LNCaP cells. The comparatively higher formation of C21 androgens can be attributed to the relative levels of the steroidogenic enzymes. In general, low CYP17A1 prohibits the formation of DHT and testosterone at higher levels. However, we observed a trend of DHT formation in 22Rv1 cells compared to the LNCaP cell lines. Because of low androsterone formation in the backdoor pathway, it is plausible that the C21 precursors in the classical pathway (e.g., progesterone, 17-OH progesterone) bypassed testosterone and forms DHT through the 5α-androstanedione pathway. The presence of CYP17A1 has been inconsistent, as reported by different research groups. CYP17A1 has been more readily detected at the mRNA level, but at the protein level, it varies from being undetectable to reaching relatively high levels when compared to other cell lines [10,21,27,36,37]. For AKR1C3, another key enzyme for testosterone and DHT production, protein levels vary similarly in LNCaP cells [22,27,29,37,38]. SRD5A1, which either converts testosterone to DHT or converts upstream steroids to the backdoor pathway, is also present in LNCaP cells [3,39,40,41]. CYP17A1, AKR1C3, and SRD5A1 are all found in 22Rv1 cells [28,29,39,42]. CYP17A1 mRNA was not detected reliably in the present study for either cell lines but CYP17A1 protein was found to be present in the past cellular experiments in our laboratory [43]. Based on the steroid precursor production and mRNA data, 22Rv1 cells do have substantially higher levels of steroidogenic enzymes for androgen production compared to the LNCaP cell line. These results are also in line with previous research investigating testosterone and DHT production in cell lines [16,44]. This is not surprising, as 22Rv1 is an androgen-independent prostate cancer cell line derived from castrated propagation conditions in mice. Thus, it was in an appropriate environment to promote de novo steroidogenesis by increasing the amount of steroid enzymes. It is interesting to note that *SRD5A1* levels are lower in 22Rv1 than in LNCaP cells. This may suggest that there are other enzymes, possibly SRD5A3, that are responsible for the conversion of steroid precursors to the backdoor pathway [12,45]. Previous reports from other laboratories have determined that SRD5A3 is overexpressed in CRPC [12,46], and its expression levels increase when androgens are depleted [47]. SRD5A3 also appears to be capable of converting testosterone to DHT [12,45,46]; however, significant future work is warranted to establish the role of SRD5A3 in steroidogenesis.

When we investigated the potential of human prostate tissue to generate steroids from upstream precursors, incubation of human prostate tissue with 17-OH-pregnenolone and progesterone produced similar trends to those observed in cell lines. There is a predominance of backdoor C21 steroid formation, along with androsterone, which is a C19 androgen. However, conversion to DHT was still limited. Interestingly, the formation of C21 steroids from the classical pathway was not stimulated. It is possible that the steroidogenesis pathway in the human prostate tissue samples may already be primed to be C21 steroid-dominant. Quantification of the steroidogenic enzymes within human prostate tissue may verify exactly how steroidogenesis pathways differ from the cellular models and whether it is consistent amongst other human tissue samples.

## 4. Materials and Methods

### 4.1. Standards, Chemicals and Reagents

5α-androstan-3α,17β-diol (3α-diol), 5α-androstan-3ß,17β-diol (3β-diol), 5α-androstan-17β-ol-3-one (DHT), 5-pregnen-3ß-ol-20-one (pregnenolone), 3ß-hydroxyandrost-5-en-17-one (DHEA), 5-androsten-3ß,17β-diol (androstenediol), 4-androsten-17β-ol-3-one (testosterone), 4-pregnen-17-ol-3,20-dione (17-OH-progesterone), 5α-androstan-3α-ol-17-one (androsterone) and 5-pregnen-3ß,17-ol-20-one (17-OH-pregnenolone) were from Steraloids Inc. (Newport, RI, USA). 4-pregnen-3,20-dione (progesterone) and 3β-Cholest-5-en-3,22-diol (22R-OH-cholesterol) were from Sigma (Oakville, Ontario, Canada). 5α-androstan-3α,17β-diol-16,16,17-d3 (d3-diol), 5α-androstan-17β-ol-3-one-16,16,17-d3 (d3-DHT) and 4-androsten-17β-ol-3-one-16,16,17-d3 (d3T) were from C/D/N Isotopes (Pointe-Claire, QC, Canada). Hydroxylamine was purchased from Fluka (Mississauga, ON, Canada). Hexane, methanol, dichloromethane, and acetonitrile were Optima^®^ grade from Fisher (Ottawa, Ontario, Canada). Chromasolv® grade ethyl acetate was purchased from Sigma and LC–MS grade formic acid was obtained from Fisher. Water was 18 MΩ purified in-house (Millipore RO unit, Billerica, MA, USA).

### 4.2. Growth and Treatment of Human Cell Lines

The LNCaP and 22Rv1 human prostate cancer cell lines were obtained from the American Type Culture Collection (Manassas, VA, USA). The LNCaP and 22Rv1 cells were authenticated by IDEXX Laboratories (Westbrook, ME, USA) and the results confirm that the genetic profiles for the cell lines were identical to the profiles reported for each cell line. LNCaP and 22Rv1 cell lines were grown and maintained in Roswell Park Memorial Institute (RPMI) medium from Gibco Life Technologies supplemented with 5% fetal bovine serum (FBS) from Hyclone. At 60–70% confluency, growth media was removed and cells were washed three times with PBS. RPMI (no phenol) supplemented with 5% charcoal stripped serum (CSS) from Hyclone was added to the cells to starve them of steroids. After 48 h, the media was replaced with RPMI (no phenol) plus 5% CSS and 2 μg/mL 22-OH-cholesterol, pregnenolone, 17-OH-pregnenolone, progesterone, 17-OH-progesterone, or no precursor for 48 h. Cell media and cell pellets were collected into glass vials and stored at −80 °C.

### 4.3. Human Prostate Cancer Tissues

Human prostate tissue (peripheral zone) samples (*N* = 9) were obtained from the Vancouver Prostate Centre tissue bank (Vancouver General Hospital, Vancouver, BC, Canada). The characteristics of the patients that have donated their prostate tissues following radical prostatectomy have been summarized in Table S1. All patients gave their informed consent before participating in the study. The study was carried out following the rules of the Declaration of Helsinki of 1975 and ethical approval (certificate #H09-01628) was procured from the Clinical Research Ethics Board (CREB) of The University of British Columbia (Vancouver, BC, Canada). Human prostate tissue samples were homogenized using the Precellys tissue homogenizer system (Bertin Technologies, Tarnos, France) as per the manufacturer′s protocol. Briefly, tissue was weighed, cross-chopped with scalpels, and homogenized in chilled 100 mM potassium phosphate plus 0.25 M sucrose buffer (pH 7.4) at a 1:7, tissue:buffer ratio using Precellys Tissue Homogenizing CKMix tubes for 10 cycles of 20 s of homogenization at 6000 rpm. The homogenate was transferred into a chilled 1.5 mL Eppendorf tube and stored in a −80 °C freezer until we were ready for the assays.

### 4.4. Steroid Extraction and Derivatization Reactions

Cell pellets were diluted 3× with water, acidified with 1/10th volume 1 M HCl, and d3T, d3Diol, and d3DHT internal standards were added prior to the extraction, which was performed twice using a mixture of hexane and ethyl acetate (70:30) at a ratio of 1:4 *v*/*v*. Media was extracted with 100% ethyl acetate at a ratio of 1:4 *v*/*v*. Extracts were dried with a Centrivap centrifugation evaporation system. Dried steroids were reconstituted and derivatized using 50 mM hydroxylamine in 50% methanol. Samples were centrifuged at 22,000 rcf for 3 min. The supernatant was collected and incubated at 65 °C for 1 h.

### 4.5. Analysis of Steroids by LC-MS

Analysis was performed on a Waters Acquity ultraperformance liquid chromatography (UPLC) coupled to a Quattro Premier XE with MassLynx^TM^ 4.1 (Waters) for instrument control. Separations were carried out with a 2.1 × 100 mm BEH 1.7 µM C18 column, mobile phase water (A) and acetonitrile (B), both with 0.1% formic acid (gradient: 0.2 min, 25% B; 8 min, 70% B; 9 min, 100% B; 12 min 100% B; 12.2 min, 20% B; 14 min run length). Column temperature was 40 °C, and injection volumes were 15 µL. The MS was set somewhat below unit resolution for enhanced sensitivity, capillary was 3.0 kV, source and desolvation temperatures were 120 °C and 350 °C respectively, desolvation and cone gas flows were 1000 L/h and 50 L/h, and the collision cell pressure was held at 6.4 × 10^−3^ mbar. All data were collected in electrospray ionization positive (ES+) mode using multiple reaction monitoring (MRM) with *m*/*z*, cone voltage, and collision energies optimized for each analyte from the scan, and fragment scan analysis of the derivatized standards. The selected transitions were the following (d3T, 307.3 > 112.1; d3DHT, 350.3 > 309.3; DHEA, 304.3 > 253.2; androstenedione, 317.3 > 124.1; 17-OH-progesterone, 361.3 > 124.1; testosterone, 304.3 > 124.1; DHT, 347.3 > 306.3; androsterone, 306.3 > 255.2; 5-pregnan-3,17-diol-20-one, 332.3 > 314.3; pregnenolone, 332.3 > 861; progesterone, 345.3 > 124.1; 5-pregnan-3,20-dione, 347.3 > 86.2; 17-OH-pregnenolone; 5-pregnan-3-ol-20-one, 334.3 > 86; 5-pregnan-17-ol-3,20-dione, 404.3 > 363.3; androstan-3,17-dione, 319.3 > 286.3). The area under the curve (AUC) of the analyte versus the internal standard was used for quantitation. Both the E and Z oxime peaks were included in integration for those steroids where separation was observed. Matrix-free calibration standards from 0.01 up to 50 ng/mL were used, without suitable blank matrix available, and the measured levels were normalized to the pellet/tissue weight or the media volume. Any matrix effect, observed primarily with greater hydrophobic analytes, was ignored with suppression considered constant across samples, and any accuracy bias was similarly consistent. A precision of within 15% was possible to at least 0.2 ng/mL for all analytes, with most being in the 0.02–0.05 ng/mL range. This level of assay characterization was considered adequate for the purposes here, allowing for good intra-and reasonable inter-analyte comparisons.

### 4.6. Steroid Biotransformation Assay with Human Prostate Homogenates

In an in vitro reaction, human prostate (peripheral zone) homogenate (45 mg/mL) was incubated with steroid substrate 17-OH pregnenolone (2 μg/mL), progesterone (2 μg/mL), or d3-testosterone (100 ng/mL, as positive control), and a NADPH-regenerating system (solution A and B) in 100 mM potassium phosphate buffer (pH 7.4), in a final reaction volume of 150 μL, at 37 °C for 60 min in a shaking water-bath. The reaction was initiated by adding NADPH, and terminated by adding 1.2 mL of chilled hexane:ethyl acetate (60:40), and d3-testosterone was used as internal standard (except for the d3T positive control). Steroids were extracted twice with hexane:ethyl acetate (60:40). Extracts were then dried down at 30 °C using a CentriVap centrifugal evaporation system, and derivatized with 50 μL of 50 mM hydroxylamine in 50% methanol (HA). HA samples were incubated at 65 °C for 1 h and analyzed by LC-MS. The levels of the designated steroids were compared to those in the T = 0 counterparts. Other appropriate controls, human liver microsomes (2 mg/mL), homogenate alone without steroid substrate, and steroid substrate alone without homogenate, were included with each assay.

### 4.7. mRNA Expression in Prostate Cancer Cells

RNA was extracted from cell pellets using TRIzol following Invitrogen′s TRIzol RNA isolation protocol. Invitrogen M-MLV and random hexamers were used to convert the RNA to cDNA. The following primers were used: SRD5A1 (forward) 5′-ACGGGCATCGGTGCTTAAT-3′, (reverse) 5′-CCAACAGTGGCATAGGCTTTC-3′; STAR (forward) 5′-GCCCATGGAGAGGCTCTATG-3′, (reverse) 5′-TTCCACTCCCCCATTGCTT-3′; HSD3B2 (forward) 5′-CGGGCCCAACTCCTACAAG-3′, (reverse) 5′-TTTTCCAGAGGCTCTTCTTCGT-3′; RDH5 (forward) 5′-GCCCGCCAGCAATGC-3′, (reverse) 5′-CGCCCAAAGCCTGAGTCA-3′; HSD17B3 (forward) 5′-TGGGACAGTGGGCAGTGA-3′, (reverse) 5′-CGAGTACGCTTTCCCAATTCC-3′; HSD17B6 (forward) 5′-ACTGCTGCCTGGTCTGCAAAGA-3′, (reverse) 5′-GAGCCACATAGTGAGGGTGCTTCC-3′; AKR1C1 (forward) 5′-GGAGGCCGTGGAGAAGTGTA-3′, (reverse) 5′-GTTGGACACCCCGATGGA-3′; AKR1C3 (forward) 5′-GGAGAAGTGTAAAGGATGCAGGATT-3′, (reverse) 5′-GTACTTGAGTCCTGGCTTGTTGAG-3. Roche SYBR green master mix was used with cDNA and primers for PCR on an ABI Viia7 real time PCR machine. Data were normalized to the level of GAPDH per sample. Primers were purchased from Integrated DNA Technologies.

### 4.8. Statistical Analyses

Differences between the mean values of two treatment groups were analyzed using the Student’s *t* test or the Mann Whitney test, and when there were more than two treatment groups, the data were analyzed by a one-way analysis of variance, followed by a Student Newman Keuls or Kruskal-Wallis multiple comparison test (SigmaStat Statistical Software, version 3.1; SPSS Inc., Chicago, IL, USA). The level of significance was set a priori at *p* < 0.05.

## 5. Conclusions

In summary, this work delineates how the steroid pathways shift towards the C21 backdoor pathway, and steroids and precursors that were used in the present study differentially affected steroid pathways. C21 steroid formation, either from the classical or the backdoor pathway, in cell lines and in the human prostate tissue model, follows a similar pattern. DHEA production in prostate cell lines is also important for maintaining androgen production. Steroidogenic enzyme levels change in response to the presence of available precursors to regulate androgen production. Human prostate tissue is more efficient than cell lines in C21 steroid formation, and 22Rv1, an androgen-independent cell line, has a greater capacity than androgen-sensitive LNCaP cells for generating DHT. Interestingly, human tissues are efficient in forming androsterone, a C19 androgen, from the backdoor pathway, which distinguishes them from the cell lines. As the first of its kind, our study demonstrates how different in vitro systems metabolize upstream precursors, and it highlights the similarities and differences in steroidogenesis pathways between the experimental systems. This will facilitate better selection of appropriate models in steroidogenesis studies during progressive adaptation and treatment resistance in PCa. Overall, due to the limited functionality of CYP17A1 in the prostate cancer models, C21 steroids are more abundant than the C19 steroids, and alternative pathways could contribute to prostatic steroidogenesis.

## Figures and Tables

**Table 1:** Steroid levels (ng/mL) detected in 22Rv1 cells after 48 h of incubation with 2 μg/mL of 22-OH-cholesterol, pregnenolone, progesterone, 17-OH-pregnenolone, 17-OH-progesterone, or vehicle. * Statistically significant (*p* < 0.05) when compared to the vehicle control. Mean ± standard error of the mean (SEM) values were obtained from three separate experiments performed on different days.

22RV1	Vehicle		22-OH-Cholesterol			Pregnenolone			Progesterone			17-OH-Pregnenolone			17-OH-Progesterone		
Cell Pellet	Avg	SEM	Avg	SEM	*p*	Avg	SEM	*p*	Avg	SEM	*p*	Avg	SEM	*p*	Avg	SEM	*p*
Dehydroepiandrosterone	0.076	0.020	0.077	0.002		1.261	0.213	*	0.292	0.043	*	15.674	6.606		0.146	0.021	
Androstenedione	0.023	0.001	0.013	0.003	*	0.029	0.013		0.327	0.179		0.022	0.008		0.510	0.183	
17-OH-Progesterone	0.223	0.110	0.249	0.219		0.078	0.046		0.994	0.094	*	6.188	0.393	*	374.535	19.418	*
Testosterone	0.059	0.001	0.061	0.005		0.018	0.005	*	0.021	0.002	*	0.058	0.008		0.082	0.014	
Dihydrotestosterone	0.068	0.041	0.038	0.006		0.098	0.060		0.050	0.009		0.057	0.007		0.176	0.104	
Androsterone	0.002	0.001	0.002	0.001		0.015	0.010		0.029	0.015		0.006	0.001		0.295	0.016	*
5-Pregnan-3,17-diol-20-one	0.000	0.000	0.012	0.012		0.000	0.000		0.156	0.127		0.295	0.028	*	101.551	29.255	*
Pregnenolone	5.249	1.113	7.483	0.258		1284.503	401.638	*	268.962	11.452	*	5.076	0.682		9.375	0.099	*
Progesterone	0.264	0.123	0.801	0.361		16.099	1.930	*	620.243	88.930	*	0.108	0.056		0.255	0.082	
5-Pregnan-3,20-dione	0.011	0.006	0.048	0.044		1.763	0.362	*	175.921	20.090	*	0.006	0.006		0.022	0.012	
17-OH-pregnenolone	0.515	0.138	0.762	0.060		14.725	3.315	*	0.590	0.205		2917.082	867.097	*	1.266	0.376	
5-Pregnan-3-ol-20-one	0.002	0.001	0.004	0.004		0.626	0.109	*	107.079	5.560	*	0.001	0.000		0.040	0.015	
5-Pregnan-17-ol-3,20-dione	0.000	0.000	0.009	0.009		0.000	0.000		0.000	0.000		0.145	0.032	*	17.217	3.558	*
5a-Androstan-3,17-dione	0.030	0.023	0.007	0.007		0.000	0.000		0.054	0.042		0.000	0.000		0.000	0.000	

**Table 2:** Steroid levels (ng/mL) detected in LNCaP cells after 48 h of incubation with 2 μg/mL of 22-OH-cholesterol, pregnenolone, progesterone, 17-OH-pregnenolone, 17-OH-progesterone, or vehicle. * Statistically significant (*p* < 0.05) when compared to the vehicle control. Mean ± standard error of the mean (SEM) values were obtained from three separate experiments performed on different days.

LNCaP	Vehicle			22-OH-Cholesterol			Pregnenolone			Progesterone			17-OH-Pregnenolone			17-OH-Progesterone		
Cell Pellet	Avg	SEM		Avg	SEM	*p*	Avg	SEM	*p*	Avg	SEM	*p*	Avg	SEM	*p*	Avg	SEM	*p*
Dehydroepiandrosterone	0.127	0.051		0.188	0.026		0.254	0.026		0.261	0.036		2.398	0.093	*	0.251	0.007	
Androstenedione	0.000	0.000		0.010	0.010		0.000	0.000		0.008	0.008		0.041	0.002		0.278	0.023	
17-OH-Progesterone	0.000	0.000		0.036	0.018		0.000	0.000		0.000	0.000		7.649	1.122	*	91.159	11.527	*
Testosterone	0.013	0.007		0.011	0.003		0.000	0.000		0.003	0.001		0.000	0.000		0.046	0.006	*
Dihydrotestosterone	0.003	0.003		0.003	0.003		0.000	0.000		0.006	0.006		0.000	0.000		0.007	0.007	
Androsterone	0.041	0.011		0.000	0.000	*	0.000	0.000	*	0.020	0.020		0.023	0.023		0.814	0.063	*
5-Pregnan-3,17-diol-20-one	0.000	0.000		0.000	0.000		0.004	0.004		0.000	0.000		0.514	0.055	*	45.665	3.027	*
Pregnenolone	3.946	1.045		5.155	0.220		1505.414	135.597	*	406.659	35.203	*	4.691	0.228		9.830	0.607	*
Progesterone	0.000	0.000		0.000	0.000		4.596	0.289	*	26.695	4.022	*	0.063	0.006	*	0.026	0.026	
5-Pregnan-3,20-dione	0.036	0.009		0.039	0.009		0.780	0.075	*	34.105	4.286	*	0.031	0.008		0.036	0.007	
17-OH-pregnenolone	0.008	0.008		0.000	0.000		0.516	0.082	*	0.016	0.016		512.809	19.425	*	0.160	0.013	*
5-Pregnan-3-ol-20-one	0.000	0.000		0.000	0.000		0.391	0.072	*	63.744	10.762	*	0.000	0.000		0.142	0.012	*
5-Pregnan-17-ol-3,20-dione	0.000	0.000		0.000	0.000		0.000	0.000		0.000	0.000		0.176	0.007	*	3.027	0.343	*
5a-Androstan-3,17-dione	0.000	0.000		0.000	0.000		0.000	0.000		0.000	0.000		0.000	0.000		0.000	0.000	

**Table 3:** A comparison of the messenger RNA (mRNA) levels of the steroid enzymes between LNCaP and 22Rv1 cells after 48 h incubation with 2 μg/mL 22-OH-cholesterol, pregnenolone, progesterone, 17-OH-pregnenolone or 17-OH-progesterone, or vehicle. Data is normalized to *GAPDH* mRNA levels, which are assumed to be constant across both cell lines. Data is represented as fold change of LNCaP levels. * represents a statistically significance (*p* > 0.05) difference between LNCaP and 22Rv1 cells.

	LNCaP	22Rv1		LNCaP	22Rv1		LNCaP	22Rv1		LNCaP	22Rv1	
	AKR1C1		*p*	AKR1C3		*p*	SRD5A1		*p*	HSD3B2		*p*
Vehicle	1	60.08	*	1	49.04	*	1	0.78		1	10.06	*
22-OH-cholesterol	1	40.88	*	1	38.98	*	1	0.69		1	9.40	*
Pregnenolone	1	59.08	*	1	66.63	*	1	0.73		1	8.77	*
Progesterone	1	18.82	*	1	69.94	*	1	0.37	*	1	10.09	*
17-OH-pregnenolone	1	41.08	*	1	62.20	*	1	0.54		1	6.77	*
17-OH-progesterone	1	18.12	*	1	40.56	*	1	0.38	*	1	8.33	*
	LNCaP	22Rv1		LNCaP	22Rv1		LNCaP	22Rv1		LNCaP	22Rv1	
	HSD17B3		*p*	HSD17B6		*p*	RDH5		*p*	STAR		*p*
Vehicle	1	11.61	*	1	9.05	*	1	31.34	*	1	4.70	*
22-OH-cholesterol	1	11.66	*	1	6.11	*	1	22.31	*	1	4.52	*
Pregnenolone	1	22.98	*	1	4.05	*	1	40.44	*	1	7.60	*
Progesterone	1	22.53	*	1	8.07	*	1	35.02	*	1	7.95	*
17-OH-pregnenolone	1	14.36	*	1	2.81	*	1	20.28	*	1	5.55	*
17-OH-progesterone	1	23.08	*	1	4.48	*	1	33.27	*	1	7.95	*

## Supplementary Materials

The following are available online at http://www.mdpi.com/2072-6694/10/10/343/s1, Table S1: Characteristics of the prostate cancer patients that underwent radical prostatectomy. Table S2: Steroid levels (ng/mL) detected in 22Rv1 media after 48 hours of incubation with 2 μg/mL of 22-OH-cholesterol, pregnenolone, progesterone, 17-OH-pregnenolone, 17-OH-progesterone, or vehicle. * Statistically significant (*p* < 0.05) when compared to the vehicle control. Mean ± SEM values were obtained from three separate experiments performed on different days. Table S3: Steroid levels (ng/mL) detected in LNCaP media after 48 h of incubation with 2 μg/mL of 22-OH-cholesterol, pregnenolone, progesterone, 17-OH-pregnenolone, 17-OH-progesterone, or vehicle. * Statistically significant (*p* < 0.05) when compared to the vehicle control. Mean ± SEM values were obtained from three separate experiments performed on different days. Figure S1: The steroidogenic enzyme mRNA fold change in 22Rv1 cells using RT-PCR after 48 h incubation with 2 μg/mL 22-OH-cholesterol, pregnenolone, progesterone, 17-OH-pregnenolone, 17-OH-progesterone, or vehicle. Values are represented as fold changes compared to the control. * represents statistically significant difference (*p* < 0.05) when compared to the control. Mean ± SEM values were obtained from three separate experiments performed on different days. Figure S2: The steroidogenic enzyme mRNA fold change in LNCaP cells using RT-PCR after 48 h incubation with 2 μg/mL 22-OH-cholesterol, pregnenolone, progesterone, 17-OH-pregnenolone, 17-OH-progesterone, or vehicle. Values are represented as fold changes compared to the control. * represents a statistically significant difference (*p* < 0.05) when compared to the control. Mean ± SEM values were obtained from three separate experiments performed on different days.
