# Supplementary material for: Characterization of Precursor-Dependent Steroidogenesis in Human Prostate Cancer Models
Source: Cancers (Basel). 2018 Sep 20;10(10):343. doi: 10.3390/cancers10100343 (PMC6210088; doi:10.3390/cancers10100343)
Supplement: Supplementary file 1 [file cancers-10-00343-s001.pdf]

Supplementary Materials: Characterization of Precursor-Dependent Steroidogenesis in Human Prostate Cancer Models

Subrata Deb, Steven Pham, Dong-Sheng Ming, Mei Yieng Chin, Hans Adomat, Antonio Hurtado-Coll, Martin E Gleave and Emma S. Tomlinson Guns

Table S1. Characteristics of the prostate cancer patients that underwent radical prostatectomy.

Sample ID[a]	Age	Prostate Weight	Pathological Gleason Score	PSA at Diagnosis	Positive for PCa	Clinical Stage
Patient 1	68	75.5	8	7.1	Yes	T2a
Patient 2	79	71.0	9	15.0	Yes	T2a
Patient 3	69	51.0	8	22.0	Yes	T2a
Patient 4	62	75.0	7	15.4	Yes	T2a
Patient 5	67	77.5	7	9.57	Yes	T1c
Patient 6	61	51	9	5.4	Yes	T2a
Patient 7	74	84	7	6.45	Yes	T1c
Patient 8	59	51	7	16	Yes	T1c
Patient 9	67	52	9	8.02	Yes	T1c

[a] All the patients had undergone radical prostatectomy as the primary treatment without any neoadjuvant therapy and did not receive any adjuvant therapy as well.

Table S2. Steroid levels (ng/mL) detected in 22Rv1 media after 48 h of incubation with 2 μg/mL of 22-OH-cholesterol, pregnenolone, progesterone, 17-OH-pregnenolone, 17-OH-progesterone or vehicle. * Statistically significant ($p < 0.05$) when compared to the vehicle control. Mean ± SEM values were obtained from three separate experiments performed on different days.

**22RV1**	**Vehicle**		**22-OH-Cholesterol**			**Pregnenolone**			**Progesterone**			**17-OH-Pregnenolone**			**17-OH-Progesterone**		
Media	Avg	SEM	Avg	SEM	*p*	Avg	SEM	*p*	Avg	SEM	*p*	Avg	SEM	*p*	Avg	SEM	*p*
DHEA	0.004	0.001	0.006	0.001		0.027	0.003	*	0.006	0.001		0.410	0.048	*	0.083	0.061	
Androstenedione	0.000	0.000	0.001	0.000		0.001	0.000		0.017	0.004	*	0.006	0.002	*	3.716	1.065	*
17-OH-Progesterone	0.000	0.000	0.000	0.000		0.005	0.000		0.118	0.048		1.809	0.165		282.437	19.036	
Testosterone	0.000	0.000	0.000	0.000		0.000	0.000		0.003	0.000		0.002	0.001		0.278	0.076	*
DHT	0.003	0.001	0.000	0.000	*	0.000	0.000	*	0.000	0.000	*	0.001	0.001	*	0.000	0.000	*
Androsterone	0.001	0.000	0.001	0.000		0.001	0.000		0.002	0.000	*	0.001	0.000		0.440	0.224	
5-Pregnan-3,17-diol-20-one	0.003	0.003	0.000	0.000		0.000	0.000	*	0.000	0.000		0.048	0.007	*	45.935	5.726	*
Pregnenolone	0.059	0.014	0.076	0.013		94.688	5.348	*	7.681	0.162	*	0.080	0.010		2.234	2.022	
Progesterone	0.000	0.000	0.001	0.000		0.818	0.184	*	71.997	25.420	*	0.003	0.001	*	0.125	0.078	
5-Pregnan-3,20-dione	0.000	0.000	0.000	0.000		0.125	0.019	*	7.268	2.477	*	0.000	0.000		0.003	0.002	
17-OH-pregnenolone	0.005	0.002	0.004	0.001		1.696	0.121	*	0.022	0.005	*	240.181	28.442	*	12.704	12.081	
5-Pregnan-3-ol-20-one	0.000	0.000	0.000	0.000		0.219	0.048	*	18.615	5.536	*	0.000	0.000		0.019	0.005	*
5-Pregnan-17-ol-3,20-dione	0.000	0.000	0.000	0.000		0.272	0.136		0.000	0.000		0.003	0.003		2.466	2.466	
5a-Androstan-3,17-dione	0.000	0.000	0.000	0.000		0.000	0.000		0.000	0.000		0.000	0.000		0.000	0.000	

Table S3. Steroid levels (ng/mL) detected in LNCaP media after 48 h of incubation with 2 μg/mL of 22-OH-cholesterol, pregnenolone, progesterone, 17-OH-pregnenolone, 17-OH-progesterone or vehicle. * Statistically significant ($p < 0.05$) when compared to the vehicle control. Mean ± SEM values were obtained from three separate experiments performed on different days.

LNCaP	Vehicle		22-OH-Cholesterol			Pregnenolone			Progesterone			17-OH-Pregnenolone			17-OH-Progesterone		
Media	Avg	Sem	Avg	Sem	P	Avg	Sem	P	Avg	Sem	P	Avg	Sem	P	Avg	Sem	P
DHEA	0.001	0.000	0.006	0.002		0.006	0.001	*	0.007	0.001	*	0.125	0.015	*	0.019	0.002	*
Androstenedione	0.000	0.000	0.001	0.000		0.000	0.000		0.002	0.000		0.006	0.001		0.330	0.117	
17-OH-Progesterone	0.000	0.000	0.000	0.000		0.000	0.000		0.001	0.001		2.817	0.224	*	77.776	10.360	*
Testosterone	0.000	0.000	0.000	0.000		0.000	0.000		0.000	0.000		0.001	0.000		0.082	0.012	*
DHT	0.000	0.000	0.000	0.000		0.000	0.000		0.000	0.000		0.000	0.000		0.000	0.000	
Androsterone	0.001	0.000	0.001	0.000		0.001	0.001		0.001	0.000		0.001	0.000		0.107	0.039	*
5-Pregnan-3,17-diol-20-one	0.000	0.000	0.000	0.000		0.000	0.000		0.000	0.000		0.060	0.004	*	6.352	0.845	*
Pregnenolone	0.010	0.001	0.015	0.001	*	44.919	6.639	*	9.277	1.296	*	0.050	0.009	*	0.058	0.010	*
Progesterone	0.002	0.000	0.002	0.000		0.173	0.011	*	15.347	2.011	*	0.002	0.001		0.016	0.004	*
5-Pregnan-3,20-dione	0.001	0.000	0.001	0.000		0.034	0.000		1.776	0.246	*	0.001	0.000		0.001	0.000	
17-OH-pregnenolone	0.000	0.000	0.002	0.001		0.020	0.002	*	0.007	0.002	*	63.400	4.638	*	0.080	0.007	*
5-Pregnan-3-ol-20-one	0.000	0.000	0.000	0.000		0.028	0.003	*	4.955	0.928	*	0.000	0.000		0.004	0.001	*
5-Pregnan-17-ol-3,20-dione	0.000	0.000	0.000	0.000		0.000	0.000		0.000	0.000		0.019	0.001	*	0.674	0.081	*
5a-Androstan-3,17-dione	0.000	0.000	0.000	0.000		0.000	0.000		0.002	0.000		0.000	0.000		0.018	0.009	

Figure S1. The steroidogenic enzyme mRNA fold change in 22Rv1 cells determined using RT-PCR after 48 h incubation with 2 µg/mL 22-OH-cholesterol, pregnenolone, progesterone, 17-OH-pregnenolone, 17-OH-progesterone or vehicle. Values are represented as fold changes compared to control. * represents statistically significant difference ($p < 0.05$) when compared to the control. Mean ± SEM values were obtained from three separate experiments performed on different days.

A

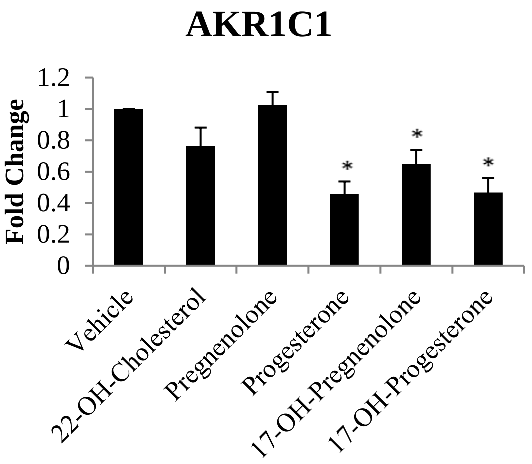

B

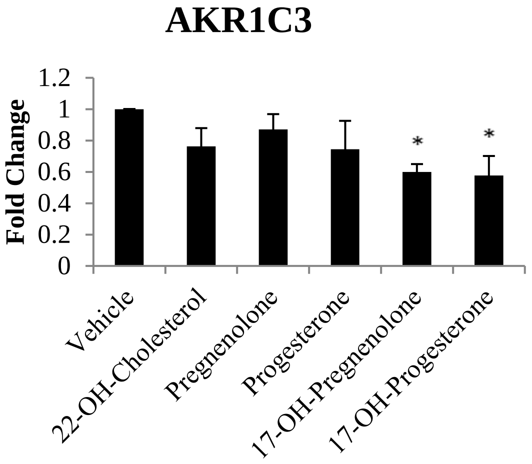

C

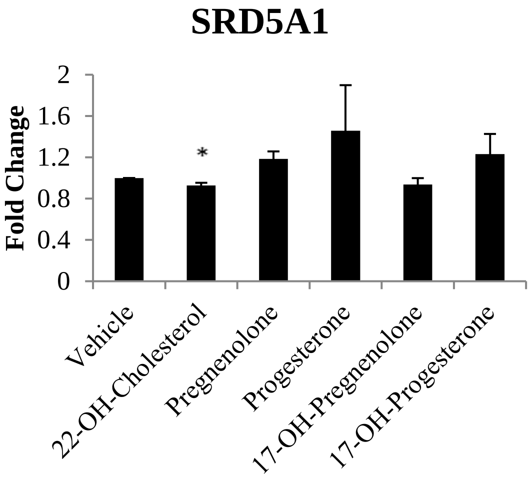

D

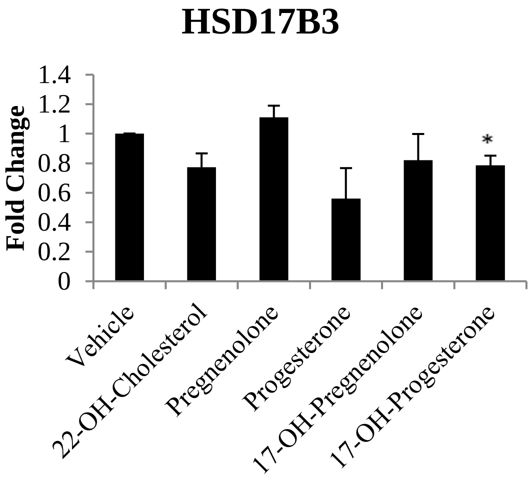

E

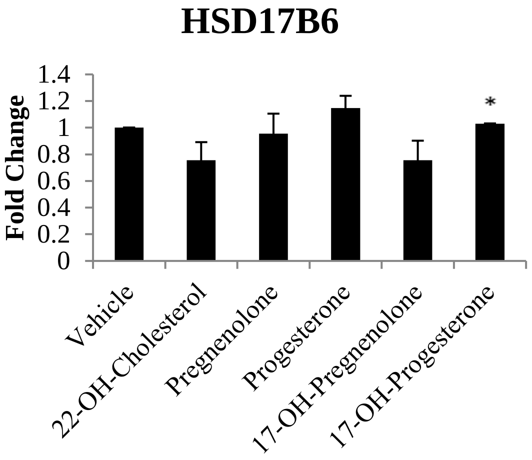
HSD17B6
Fold Change
1.4
1.2
1
0.8
0.6
0.4
0.2
0
*
Vehicle
22-OH-Cholesterol
Pregnenolone
Progesterone
17-OH-Pregnenolone
17-OH-Progesterone

Figure S2. The steroidogenic enzyme mRNA fold change in LNCaP cells determined using RT-PCR after 48 h incubation with 2 μg/mL 22-OH-cholesterol, pregnenolone, progesterone, 17-OH-pregnenolone, 17-OH-progesterone or vehicle. Values are represented as fold changes compared to the control. * represents statistically significant difference ($p < 0.05$) when compared to the control. Mean ± SEM values were obtained from three separate experiments performed on different days.

A

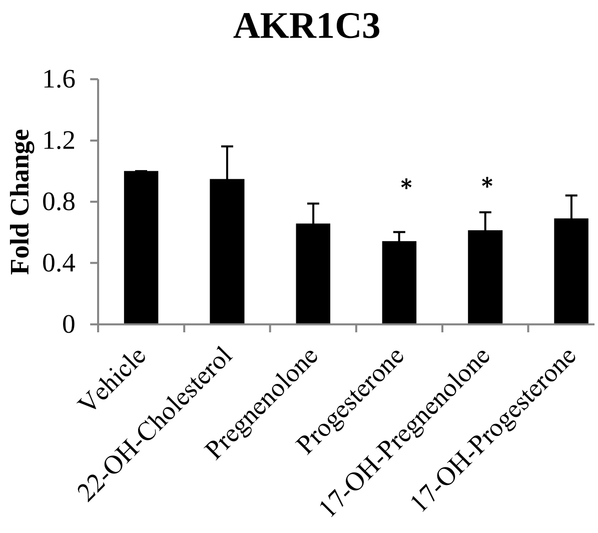

B

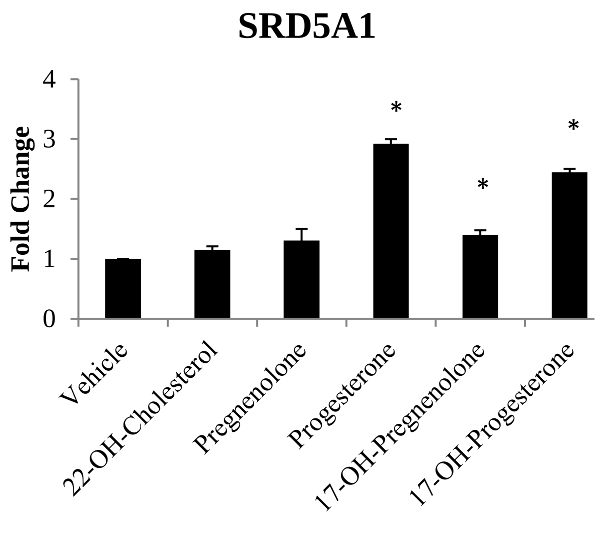

C

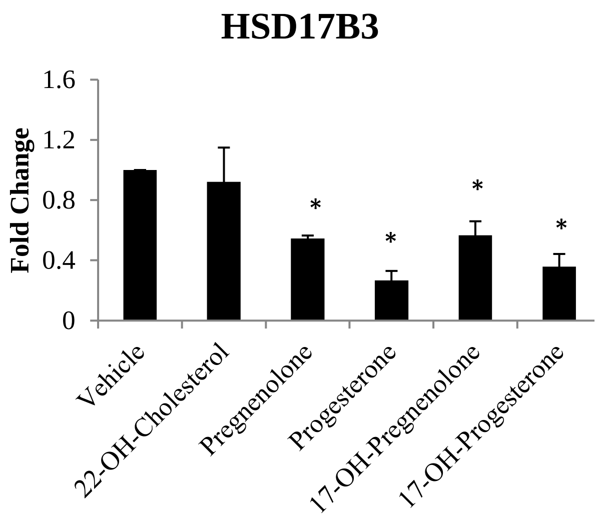

D

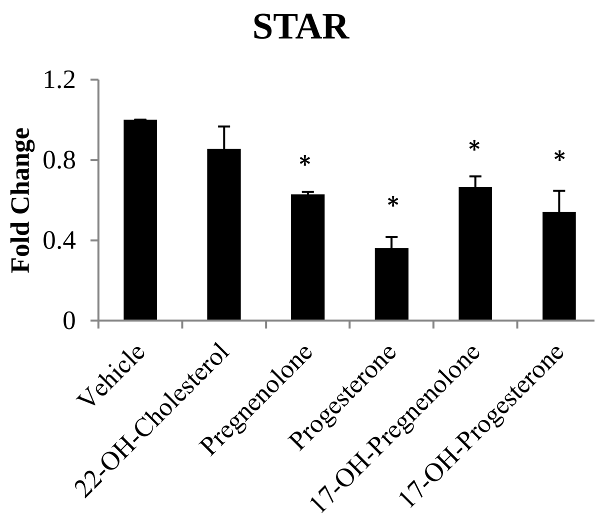

© 2018 by the authors. Licensee MDPI, Basel, Switzerland. This article is an open access article distributed under the terms and conditions of the Creative Commons Attribution (CC BY) license (http://creativecommons.org/licenses/by/4.0/).
